# Cognitive and Physical Intervention in Metals’ Dysfunction and Neurodegeneration

**DOI:** 10.3390/brainsci12030345

**Published:** 2022-03-03

**Authors:** Anna Jopowicz, Justyna Wiśniowska, Beata Tarnacka

**Affiliations:** 1 Department of Rehabilitation, Eleonora Reicher National Institute of Geriatrics, Rheumatology and Rehabilitation, Spartańska 1, 02-637 Warsaw, Poland; just.wisniowska@gmail.com; 2 Department of Rehabilitation Medicine, Faculty of Medicine, Warsaw Medical University, Spartańska 1, 02-637 Warsaw, Poland; btarnacka@wum.edu.pl

**Keywords:** metals, neurodegeneration, cognitive rehabilitation, physical rehabilitation, neurodegenerative disorders

## Abstract

Metals—especially iron, copper and manganese—are important elements of brain functions and development. Metal-dysregulation homeostasis is associated with brain-structure damage to the motor, cognitive and emotional systems, and leads to neurodegenerative processes. There is more and more evidence that specialized cognitive and motor exercises can enhance brain function and attenuate neurodegeneration in mechanisms, such as improving neuroplasticity by altering the synaptic structure and function in many brain regions. Psychological and physical methods of rehabilitation are now becoming increasingly important, as pharmacological treatments for movement, cognitive and emotional symptoms are limited. The present study describes physical and cognitive rehabilitation methods of patients associated with metal-induced neurotoxicity such as Alzheimer’s disease, Parkinson’s disease, amyotrophic lateral sclerosis, Huntington’s disease and Wilson’s disease. In our review, we describe physical (e.g., virtual-reality environments, robotic-assists training) and psychological (cognitive training, cognitive stimulation, neuropsychological rehabilitation and cognitive-behavioral and mindfulness-based therapies) methods, significantly improving the quality of life and independence of patients associated with storage diseases. Storage diseases are a diverse group of hereditary metabolic defects characterized by the abnormal cumulation of storage material in cells. This topic is being addressed due to the fact that rehabilitation plays a vital role in the treatment of neurodegenerative diseases. Unfortunately so far there are no specific guidelines concerning physiotherapy in neurodegenerative disorders, especially in regards to duration of exercise, type of exercise and intensity, as well as frequency of exercise. This is in part due to the variety of symptoms of these diseases and the various levels of disease progression. This further proves the need for more research to be carried out on the role of exercise in neurodegenerative disorder treatment.

## 1. Introduction

Metals are important trace elements in plants and animals. Unfortunately, an excess of metals may accumulate in various human organs, including the brain. Elevated levels of metals can be related to various harmful intracellular processes such as oxidative stress, DNA fragmentation, mitochondrial dysfunction, endoplasmic reticulum stress, autophagy dysregulation and apoptosis induction [1]. These processes can affect neurotransmission, leading to neurodegeneration with cognitive problems, movement disorders and learning and memory dysfunction. It has been proven that metal-induced neurotoxicity is associated with multiple neurological diseases such as Alzheimer’s disease (AD), Parkinson’s disease (PD), amyotrophic lateral sclerosis (ALS), autism spectrum disorders (ASDs), Guillain–Barré syndrome, Huntington’s disease (HD), manganism, Gulf War syndrome (GWS), multiple sclerosis (MS) and Wilson’s disease (WD), as well as neurodegeneration with brain iron accumulation (NBIA) [1,2,3,4,5]. 

Rehabilitation, according to the World Health Organization (WHO), is defined as “a set of interventions designed to optimize functioning and reduce disability in individuals with health conditions in interaction with their environment”. Neurological rehabilitation helps patients to overcome movement and cognitive impairments and helps in returning patients to more normal participation in daily life. Physical training and exercise (PE) can improve mental status, physical performance and health in general. Training may be augmented by virtual reality environments, robotic assists or noninvasive brain stimulation. All such procedures should promote neuronal survival and neuroplasticity. There is accumulating evidence that moderate-to-vigorous physical activity has many benefits to brain health and cognitive function [6,7,8,9,10,11,12,13,14]. Physical activity decreases the risk of dementia (AD and PD); may be useful in mood abnormalities as depression; can diminish stress and anxiety; and has a positive effect on memory, attention, learning, etc. [15,16]. Copper and iron are essential trace elements for the human body since they act as a cofactor of several enzymes and proteins and play a pivotal role in several biological functions such as respiration and protection from oxidative damage; and in the central nervous system, functioning myelination synthesis of neurotransmitters, neuropeptides activation, etc. Their dysmetabolism is associated with different toxic effects, mainly represented by oxidative stress, and has been reported in many neurodegenerative disorders such as neurodegeneration with NBIA, including kinase-associated neurodegeneration (PKAN), Wilson’s disease, Menkes disease, AD, PD and ALS [3,17,18,19]. In AD, an excess of iron and iron-metabolism dysregulation is involved in amyloid-beta (Aβ) production and aggregation, causing neuronal cell death [20]. 

AD and PD are among the most common neurodegenerative diseases, with motor and psychobehavioral dysfunction symptoms. Its pathogenesis is very complex and not fully understood. Neurodegeneration is a functional and/or structural loss of neurons in the central nervous system, leading to cognitive and motor decline. The abnormal accumulation of (Aβ) and tau proteins, as well as alpha-synuclein or transactive-response DNA-binding protein, is the most common histopathological process leading to neurodegeneration [21]. Neuroinflammation with glial cells and astrocyte accumulation potentiate further neural injury [22]. AD is a type of amyloidosis with amyloids depositing in the neurons and glia; it is also a tauopathy, and tau protein is considered secondary to the deposition of Aβ. Amyloid-β deposits may trigger Ca^2+^ flux through the plasma membrane and increase intracellular Ca^2+^ concentration. This leads to mitochondrial Ca^2+^ overload, oxidative stress and pro-apoptotic mitochondrial protein production [23]. In PD, the most characteristic pathomorphological process is the presence of Lewy bodies (LBs) with abnormal aggregates as α-syn. The TDP-43 protein is a major component which aggregates in ALS. It has been found in ALS patients in the spinal cord, brainstem and motor cortex [21]. In all these neurodegenerational disorders, metal dyshomeostatis is proposed as a pathological pattern leading to neurodegeneration [5,19,24]. Iron and copper can induce oxidative stress and may stimulate DNA damage and the mitochondria and lipid membranes’ impairment. The metal ions’ location within central-nervous-system cells suggests their role in the pathogenesis of neurodegenerative conditions such as AD, PD, ALS and glial tumors [25]. It has been proposed that in AD, copper may lead to amyloid fibril accumulation [26]. In animal models, the use of copper-chelating agents may reverse amyloid deposits in the brain and prevent oxidative stress in senile plaques and neurofibrillary tangles, which can support the role of copper in the etiopathogenesis of AD [27]. In the quantitative susceptibility map, the colocalization of brain iron and Aβ plaques was confirmed, and showed that the colocalization of brain-iron deposition and Aβ plaques promoted the development of AD [28]. There is also evidence of iron accumulation and Aβ aggregation using transmission electron microscopy [29]. Recently, new evidence has suggested that copper in AD pathology may be related to neuroinflammation. It has been shown that ceruloplasmin—the major copper-transporting protein in plasma and copper-containing ferroxidase—was elevated in the brain and serum in AD patients, which can induce proinflammatory responses in cultured microglia [30]. The intracellular excess of iron in the aging process may further exacerbate inflammation in AD and promote the production of amyloid-precursor protein through the iron-reactive element in its promoter [31]. There is also growing evidence that the accumulation of several metals, including iron, copper and manganese, in substantia nigra may play a causative role in the pathophysiology of PD [32]. Many facts and mechanisms may be involved in this process, as epidemiological studies suggest a relationship between environmental exposure to metals and the risk of PD, as it has been observed that disorders with brain-metal accumulation may manifest with parkinsonism; in animal studies, the effects of chelation treatment were detected, and measurements of metal concentrations and metal regulatory protein expression in cerebrospinal fluid and post-mortem brain-tissue samples [32]. ALS pathology also appears to include metal imbalances, and a known risk factor for ALS can be environmental-metal exposure [33]. It has been proven that administration of a Cu-chelating agent (ammonium tetrathiomolybdate) enhanced survival and slowed ALS progression [34].There is accumulating evidence that exercise can enhance brain function and attenuate neurodegeneration in such mechanisms as improving neuroplasticity by altering the synaptic structure and function in many brain regions. There is also evidence that exercise modulates multiple systems that are known to regulate neuroinflammation and glial activation [6]. Activated microglia and proinflammatory cytokines play a pivotal role in the pathogenesis of neurodegenerative diseases such as AD and PD [6,13,35]. There is a body of evidence that exercise is felt by the brain, suggesting that there is feedback between muscle and brain function [7]. During exercise, skeletal muscles communicate with other organs by producing and releasing so-called myokines. Myokines are secreted from muscle cells during differentiation and proliferation and/or in response to muscle contractions. Muscles have been shown to secrete myokines that contribute to the regulation of hippocampal function [36,37]. There is evidence that the myokine cathepsin B can cross the blood–brain barrier leading to increased production of brain-derived neurotrophic factor, thereby affecting neurogenesis and learning and memory [14,36,37,38]; exercise may increase neuronal expression of the FNDC5 gene encoding the PGC1α-dependent myokine FNDC5, which subsequently may result in increased levels of neurotrophic factor [7]. Finally, exercise also may reduce depression-like symptoms via multiple mechanisms, as well as via the mechanism of increasing the PGC1α-dependent muscular expression of kynurenine aminotransferase enzymes, which can induce a beneficial effect in the balance between the neurotoxic kynurenine and the neuroprotective kynurenic acid [7].

Studies in animals have also shown that adiponectin can also be expressed and released from muscles in association with exercise, which can have a beneficial impact on cognitive function and neurogenesis [39]. Exercise has also been shown to have a positive impact on learning, memory and attention, as well as for executive functions, reaction time and language learning, motor skills learning, verbal and visuospatial cognitive functions, visual figure recognition, spatial memory and attentional control [40,41,42,43,44,45]. 

Iron-dysregulation homeostasis is well-known and evident in AD [2], but there is still little known about the influence of physical exercise on iron metabolism in the brain and peripheral nervous system, as well as the mechanisms responsible for exercise-induced iron regulation in AD. In the animal model, long-term voluntary running induced the redistribution of iron, which had an impact in altered iron metabolism and trafficking in the brain, and increased iron content in skeletal muscle [46]. Belaya et al. found that exercise reduced levels of cortical hepcidin, a key regulator of iron homeostasis, and reduced the levels of interleukin-6 (IL-6) in cortex and plasma [46]. The authors stated that regular exercise can induce a reduction in hepcidin in the brain, possibly via the IL-6/STAT3/JAK1 pathway [46]. 

Metal deposition and neurodegenerational diseases are associated with damage to brain structures associated with the wider motor system. The majority of pathologies in PKAN—the most commonly recognized set of symptoms from NBIA—are found in the globus pallidus and variably in adjacent structures [47]. It has later consequences in imaging studies, as the “eye of the tiger” sign identified on magnetic resonance imaging (MRI) images correlates to a region of rarefaction in the center of the globus pallidus. Iron deposits can be also seen in in the white and gray matter of the cerebrum and other structures [48]. Recently it was suggested that tissue or cellular hypoxic/ischemic injury in the globus pallidus may underlie the pathogenesis of PKAN, when apolipoprotein E enrichment in ubiquitinated proteinaceous aggregates in the globus pallidus in patients with PKAN were seen [49]. In WD—a representative of diseases in the circle of disorders related to the deposition of copper in the brain—different brain regions have distinct susceptibility to copper toxicity. The most reported pathological changes in the basal ganglia, thalamus, cerebellum and upper brainstem are reported [50], which is also related to abnormalities on (MRI). The putamens are the most frequently affected brain region in WD, with lesions linked mainly to dystonia and parkinsonism, cerebellum with ataxia, etc. 

Neurological signs and symptoms of classic PKAN, WD or neuroferritinopathy are primarily extrapyramidal and include dystonia and dysarthria, tremor, parkinsonism, gait and posture disturbances. Corticospinal tract involvement can also be seen in WD and PKAN with spasticity, hyperreflexia and extensor toe signs. Seizures are quite rare. Chronic manganese (Mn) exposure can also cause debilitating neurological effects. Mn overexposure can lead to parkinsonism, known as manganism, and it has been suggested that it may also be a part of PD etiology [51]. Manganism is characterized by different tremors and speech impediments and lethargy, with the occasional accompaniment of psychosis. Psychiatric symptoms are common signs in disorders with metal accumulation, and can also precede neurological signs or be the first sign of disease [24,52]. Intellectual impairment is recognized in PKAN, particularly in children with young onset. Dementia is a common sign in aceruloplasminemia; mood disturbances are the most common psychiatric manifestation of WD [50]. 

Physical and occupational therapy and cognitive therapy should be indicated in metal-accumulation disorders, particularly for those who are only mildly symptomatic. Therapies to maintain normal joint mobility for as long as possible may be useful. In cases with gait impairment, some orthosis or other kind of aids are needed (e.g., a walker or wheelchair for gait abnormalities). Speech therapy and/or assistive communication devices are needed for PKAN or WD and other diseases with dysarthria and speech delay. 

## 2. Psychological Methods of Treatment of Neuropsychiatric Disorders Associated with Storage Disease 

Patients suffering from storage disease usually exhibit cognitive and emotional disorders. Standard psychological methods of treatment are now becoming increasingly important as the pharmacological treatment for cognitive and emotional symptoms is limited. The psychological methods used primarily focus on improving patients’ general functioning and quality of life, as well as supporting their independence. If the disorders are related to episodic memory function and learning disability (for example, AD), functional cognitive therapy might be more helpful than psychotherapeutic interventions. In diseases where memory is relatively preserved until the later disease phases (PD, HD, ALS), psychotherapeutic interactions may be more applicable in early interventions. 

The most important methods supporting cognitive impairment are cognitive training, cognitive stimulation and neuropsychological rehabilitation [53]. Exercises of specific cognitive domains are incorporated into cognitive training. Further, cognitive stimulation means general support of cognitive functions using reminiscence therapy (referring to the facts and history of the patient’s life with the use of familiar pictures, music, films, etc.) and reality orientation training (repeating the same information in short time intervals, especially in reference to that very person and the time and place of meeting) [54]. Neuropsychological rehabilitation uses special cognitive exercises adapted to the patient’s needs and possibilities aimed at a specific goal. The most common cognitive problems associated with storage disease are memory, executive, visual-spatial, attention, language functions and psychomotor speed, social skills and self-consciousness impairment [55]. The choice of the type of cognitive therapy depends on the depth of the cognitive disorder. In mild cognitive disorders, cognitive therapy and neuropsychological rehabilitation should be recommended [56]. In moderate-to-serious cognitive disorders, especially memory impairment, cognitive stimulation (reminiscence therapy and reality orientation training) can be proposed [57]. An example of nonpharmacological methods of treating emotional disorders is psychotherapy, which applies to comorbid depression and anxiety-symptom treatment [53,58]. Psychiatric symptoms are mainly associated with the process of brain damage; however, a lot of symptoms are in fact a reaction to the progressive disability and the awareness of the problem. Efficacy in contemporary literature refers to cognitive-behavioral (CBT) and mindfulness-based (MBTs) therapies. CBT is an intervention that addresses maladaptive thinking and behavior patterns related to the onset and maintenance of various psychiatric disorders, including depression [59]. MBTs involve ‘paying attention in a particular way: on purpose, in the present moment, and non-judgmentally’ [60]. 

Here we present the newest data concerning possibilities of physical and psychological rehabilitation in patients with brain-metal-accumulation disorders. 

## 3. Physical Activities in Treatment of Neuropsychiatric Disorders Associated with Storage Disease 

There are currently no specific guidelines in regard to which forms of physiotherapy should be used in the treatment of patients with the aforementioned neurodegenerative diseases. Symptoms that most often affect manual dexterity in patients with these diseases and require physiotherapy include bradykinesia, gait and balance disorders, dystonia and ataxia. 

The ideal combination of interventions for each patient is dependent on the stage of disease progression, patients’ preferences regarding type of exercise, patients’ learning ability and age [61].

Due to the fact that bradykinesia is a result of impaired central drive, exercise interventions should be aimed at improving the speed, amplitude and pacing of both the limb and body center of mass movements. These exercises enhance weight shift and postural control and include exercises such as lunges, kicks and quick boxing movements. Other types of exercise used involve patients taking large steps while tilting beyond their stability limits and while responding to attempts at external displacement, such as those involving hitting a punching bag. Walking sticks could be useful in increasing patients’ attention to large, symmetrical arm swings coordinated with strides [62]. Ni et al. demonstrated that the 3-month power-training program (PWT) had a significant impact on the alleviation of bradykinesia, as well as on improving muscle strength and power in older patients with PD. PWT has been shown to be effective in improving physical function, and as such, quality of life [63]. Xu et al. proved the superiority of PWT over conventional resistance training in improving physical functions such as balance, muscle strength and walking speed [64].

Gait impairments, alongside bradykinesia, are some of the most common and debilitating symptoms of neurodegenerative diseases, and additionally exacerbate the patients’ risk of falling. The beneficial effects of exercise in reducing motor symptoms resulting from neurodegenerative diseases have been extensively documented. These include better posture and balance control, as well as improved physical condition [65]. Gait parameters such as velocity, stride and step length have been shown to improve thanks to treadmill training, thus proving its efficacy as a means of gait training [65]. The practice of functional activity due to it being a task-specific form of training could result in a meaningful improvement. This form could be used during training interventions focused on improving walking or grasping. In addition, turning-based forms of training improve vestibular integration ability in PD patients. Enhanced balance ability and muscular strength reduce the risk of falls in this patient population. Task-specific training is proven to be more effective than traditional exercise in improving functional performance. It has also been demonstrated that curved-walking training (CWT) considerably improves walking speed, cadence and step length in comparison with the effects of general exercise [66]. Additionally, a positive impact that lasted over a month on FOG was also observed, thus further proving the superiority of task-specific training over traditional general exercise [64]. In the early stages of PD, exercises reducing the risk of freezing should be performed in situations where freezing often occurs. These exercises typically involve skipping, high stepping, taking large steps in varying directions, as well as over and around obstacles, situated shoulder-width apart. Other exercises could involve practicing quick turns around corners and near walls. Such exercises may reduce freezing episodes. Patients with PD should partake in such exercises in a home environment or gym where the appropriate obstacle courses have been set up. These obstacle courses should include activities requiring negotiating through narrow and tight spaces, stepping over hurdles, picking objects off the floor while walking, swiftly switching directions and foot placement [62]. Dual-task aquatic exercise programs have shown promise in aiding the therapy of patients with PD due to the potential to enhance functional mobility, balance and gait in these patients [67]. 

One of the most common symptoms of PD and other neurodegenerative diseases is axial rigidity, which results in a loss of natural vertebral, pelvis/shoulder girdle, and femur/pelvis flexibility and a range of motion that accompanies efficient postural and locomotor activities.

King and Horak recommended an exercise program that encompasses reciprocal movements, improves axial rotation, stretches flexor muscles and strengthens extensor muscles in order to facilitate an erect posture [62]. Kayaking could aid in a reduction in rigidity, as it is a form of exercise involving counter rotations of the shoulders and pelvic girdle. Exercises that have a similar effect on rigidity include tai chi, during which focus is placed on the patient’s perception of alignment during postural transitions; as well as pre-pilates, which focuses on enhancing spinal mobility and lengthening flexor muscles. Additionally, the exercise program should incorporate exercises that improve head and trunk rotation through turning, switching from standing to sitting position, and vice versa [62]. 

The full extent of the effects that PA and exercise engagement have on the physical and psychological health of patients with dystonia is as yet not fully understood. Specific guidelines are required to deal with this. Studies should also investigate other disorders connected with dystonia, as well as the benefits of a holistic approach to treatment through multidisciplinary clinical trials assessing patient-reported outcome measures [68]. 

The fact that patients with mild ataxia in comparison to those with moderate-to-severe ataxia are more capable of motor learning means that they are more likely to experience the benefits of focused physical therapy. This physical therapy is aimed at enhancing postural balance and gait. Included in the program are exercises to improve general conditioning, increase muscle strength and expand the range of motion in the trunk and limbs, as well as exercises aimed at improving static and dynamic balance in a wide variety of positions. Exercises to boost ADLs, relaxation, the ability to perform basic hygiene related routines, balance and performance of dual-motor tasks should be included in the occupational therapy program [69]. In cerebellar ataxia, physical therapy programs usually focus mainly on the use of balance exercises—both static and dynamic—and on coordination exercises. 

The use of virtual reality tools in this field also shows real potential. Usage requires balance and general coordination and the game-based aspect means patients are more likely to adhere to treatment. 

Biofeedback could be beneficial in programs to improve gait and balance. Gait training on treadmills could also be helpful [70]. 

A number of symptoms in patients with movement disorders such as PD or dystonia have been treated with the use of deep brain stimulation (DBS). While ataxia syndromes are not generally treated with DBS, patients with ataxia and dystonia or drug-induced refractory tremors could benefit from this therapy. It is worth noting that while DBS could potentially be used to treat tremors in patients with underlying ataxia, there is an attendant risk of inducing exacerbation of gait disorders and other symptoms [71].

As mentioned above, treatment methods such as virtual reality, biofeedback and treadmill training with supported body weight and torso weighing exhibit potential to be effective, but their specific efficacy is currently yet to be defined. This work is limited by the large number of underlying conditions studied, as well as methodological failings such as small sample sizes, inadequate descriptions of rehabilitation protocols, etc. Published data from large, randomized trials is also unavailable. Additional research is of utmost importance to ascertain optimal dosage, duration and intervals of therapy needed to achieve appreciable functional improvement [70]. 

Dysphagia is one of the most debilitating symptoms experienced by patients with PD. Oropharyngeal dysphagia is present in up to 80% of patients with early-stage PD [72,73,74]. Incidence of this symptom rises up to 95% in advanced stages of the disease. In spite of this, there is inadequate confirmation of the efficacy of interventions used in the treatment of dysphagia in PD patients. The most frequently used current treatment method is aimed at making changes in regard to the texture of food; however, there are also other techniques focused on enhancing the quality of life of these patients. Such techniques include rehabilitative therapies such as EMST (expiratory muscle strength training) or NMES (neuromuscular electrical stimulation) and have been effective at improving swallowing and oropharyngeal function while reducing the risk of choking and aspiration [73]. 

A study by Smith, Roddam and Sheldrick reviewed literature on both compensatory and rehabilitation intervention techniques for dysphagia in PD patients. They argued that rehabilitation methods show immense possibility to improve swallowing safely, and as such, improve quality of life [75]. Gadenz et al. in their review discussed the benefits of repetitive transcranial magnetic stimulation (noninvasive transcortical stimulation) in relation to neurogenic disorders involving communication and swallowing function in stroke patients; however, findings are inconclusive [76]. Compensatory techniques have been more extensively researched than rehabilitation interventions, but evidence is as yet undetermined. After the implementation of aforementioned techniques, improvements were observed in regards to degenerative function (coordination, speed and volume), quality of life and social interaction of patients with PD. As earlier discussed, further research is essential to develop a clinical decision-making model for treatment methods for these patients. Examples of exercises in neurodegenerative diseases are presented in Table S1.

## 4. Selected Metal Storage Diseases and Cognitive and Motor Rehabilitation 

### 4.1. Alzheimer’s Disease

AD is the leading cause of cognitive impairment and dementia in people over the age of 65. Assessment of older patients with cognitive and behavioral symptoms or functional decline should encompass the use of a structured history, symptom-function evaluation from patients and carers, as well as running a variety of tests including laboratory tests and neuroimaging. Evaluations of in vivo biomarkers such as cerebrospinal fluid and PET are also available [77]. Typical neuropsychological screening tools recommended in AD are the mini-mental state examination (MMSE) and the clock-drawing test (CDT). In conditions other than AD dementia, the Montreal Cognitive Assessment (MoCA) and the Mini-Addenbrooke’s Cognitive Examination-III (Mini-ACE-III) are recommended [78]. 

The neuropsychological diagnosis includes functional assessments in many cognitive domains. There are no specific tests recommended for diagnosing different types and variants of dementias [79]. For example, regarding diagnosis, the Digit Span from the Wechsler Intelligence Scale for Adults and the (color) Trail Making Test can be used. For the memory assessment, the California Verbal Learning Test and the Rey Complex Figure Test can be helpful. Executive functions can be examined by using the verbal fluency test, the Stroop test and the Rey Complex Figure Test. A visual-spatial ability diagnosis typically involves the Rey Complex Figure Test, whereas language skills are tested by picture naming and the use of the verbal fluency test [80].

#### 4.1.1. Physical Methods of Rehabilitation 

A sedentary lifestyle is known to be a possible risk factor for the earlier onset of AD [81]. Exercise as a nonpharmacological strategy could help protect against cognitive decline and reduce the risk of AD [82]. Physical exercise also delays the onset of severe neuropsychiatric symptoms such as apathy, confusion and depression in patients with AD [83].

Several studies suggest that exercise has the potential to improve sleep in persons with AD. However, there is a lack of RCTs or observational studies investigating the relationship between physical activity and polysomnographically measured sleep outcomes in this patient population. As such, more research evaluating the influence of environmental, interpersonal or physical attributes of patients and caregivers in order to develop the most ideal physical activity programs to optimize sleep, motor and cognitive outcomes in patients with NDD is much-needed [84].

Recent studies show that exercise interventions are capable of attenuating the symptoms of neurodegeneration experienced by AD patients, with comparable observations in rodent models of AD [81].

As exercise can aid in reducing the occurrence of dementia and AD, it is often cited as a possible lifestyle intervention. While studies have demonstrated the potential benefits of exercise in regards to reducing the rate of declining cognition, evidence is still lacking concerning the benefits of exercise in patients with AD specifically. Some of the previous studies have limitations associated with randomization and surveillance in the group with treatment. There are also relatively few large-scale studies focusing on Alzheimer’s patients [85,86].

Due to this limitation, it is difficult to formulate clear health instructions about the best types/combinations of types of exercise, as well as the intensity, frequency and duration of said physical activity required to reduce the risk or delay the onset of AD.

In general, exercise therapy is a viable, cost-effective method of treatment that has the potential to improve cognitive function, slow down rapid cognitive impairment and delay progression to dementia in older patients at risk of dementia [54]. 

#### 4.1.2. Cognitive Therapy 

In AD, the most common psychological method refers to cognitive impairment, especially episodic memory decline. The reports about the efficacy of psychotherapy in AD mostly refer to patients’ caregivers’ problems, rather than patients’ psychiatric disorders [87]. Useful cognitive therapy methods include reminiscence therapy, special cognitive rehabilitation program and cognitive training [88,89]. An example of cognitive training addressed to AD patients, described by Brueggen et al. [88], included six modules with two sessions each, for a total of 12 meetings. Subsequent modules concerned the identification of problems and definition of treatment goals, the use of external memory aids, introduction and implementation of daily routines and a structured framework for the day, organization and implementation of pleasurable and meaningful activities, reminiscence and biographical work, evaluation of achieved goals and planning of future procedures [88]. 

The current literature shows a general improvement in functioning in several domains in patients with mild-to-moderate AD after cognitive interventions. For example, reminiscence therapy might be effective in improving global cognitive functioning and quality of life and reducing depression symptoms [57]. Cognitive training can improve verbal initiative and stabilize memory, while noncognitive (for example, music therapy and education) treatments can ameliorate psychosocial aspects [89,90]. Moreover, even if the cognitive training does not improve cognitive functioning, it can contribute to an improvement in quality of life and the maintenance of (the level of) self-reliance/independence [88]. In other words, even when global cognitive functioning was declining, everyday life independence remained on the same level in patients with AD after cognitive training [91]. Future research might be focused on the interactions between cognitive training and another supporting conventional method; for example, transcranial direct-current stimulation [92].

### 4.2. Parkinson Disease

Initially, PD was strictly regarded as a movement disorder characterized by its three main motor symptoms of bradykinesia, rigidity and tremors. However, the occurrence of other additional motor and nonmotor symptoms have led to the revision of clinical diagnostic criteria. The new criteria, other than highlighting the three cardinal motor signs, also define supportive criteria, absolute exclusion criteria and red flags. 

Absolute exclusion criteria include cerebellar abnormalities, supranuclear gaze palsy, frontotemporal cognitive impairment, lack of response to levodopa treatment, slow progression and cortical symptoms such as apraxia, as well as a normal DAT scan. 

Red flags include early gait impairment, lack of disease progression, inspiratory respiratory dysfunction, advanced autonomic failure, especially in the first year of disease, pyramidal tract symptoms, early antecollis, bilateral and symmetrical parkinsonism and the absence of common nonmotor signs that occur in PD such as hyposmia, autonomic dysfunction or sleep disorders [93]. 

The nonmotor symptoms (NMS) in PD include pain, fatigue, low blood pressure, restless legs, bladder, bowel, skin and sweating problems, sleep disturbances, eating and swallowing dysfunctions and emotional and cognitive impairment [94].

Clinimetrics, assessment of balance and gait, arm/hand function and gait/walking are used in the motor assessment of PD. The most commonly used clinimetric scales used are the Hoehn and Yahr stages of disease progression and the Unified Parkinson’s Disease Rating Scale. Others used in the assessment of balance and posture include the Berg BS, Brunel BA, Tinetti and TUG tests. 

Some of the most commonly known skill tests are the Purdue Pegboard Test, Nine-Hole Peg Test, Pig-Tail Test, Frenchay Arm Test, Jebsen and Taylor Test, Wolf FMT and Finger-tapping Test.

Popular motricity scales include the Fugl-Meyer Motor Assessment Scale and the Södring Motor Evaluation. Quantitative and qualitative assessments of gait and balance are also used [95].

#### 4.2.1. Physical Methods of Rehabilitation 

##### Aerobic Exercise Training (AET)

Aerobic exercise training (AET) is generally regarded as the best choice of exercise program for health improvement [65,96]. 

AET has been proven to positively impact patients’ walking capacity, exercise function, velocity and step length. A number of clinical studies have shown that continuous exercise improves the ability to carry out daily activities in regards to bradykinesia, balance and turning in patients with early-stage PD [65]. Such exercise interventions have also been found to improve general health-related quality of life (HRQL) in patients with PD [97]. Due to the positive effects on motor function, cognition and emotions, AET is a very popular treatment choice for function recovery in PD patients. 

One form of AET that, in addition to being safe, also improves aerobic ability, motor performance and cognitive function in early-stage PD patients is stationary-bicycle exercise [98,99]. Cycling is also an interesting choice for people with freezing of gait (FOG) [100]. It has been proven that regular moderate AET has a positive effect on depression and anxiety. In comparison with pharmacological therapy, exercise was equally effective in the treatment of depression after a period of treatment lasting 16 weeks. Additionally, patients who did not respond to antidepressant medication felt better after AET (30–45 min. sessions, 5 days per week) [101]. Functional MRI reward task imaging showed that regular aerobic exercise could enhance ventral striatum activity in PD patients, thus suggesting that exercise possibly participates in the activation of the mesolimbic pathway, and the increased capacity to anticipate rewards may contribute to mood improvement [102].

In comparison with other forms of exercise such as Qigong and Tai chi, AET is reported to be the most effective for alleviating depression in PD patients [103]. 

Exercise intensity also influences the level of cognitive performance improvement in PD patients. This was demonstrated in a comparison between the effects of high-intensity interval training (HIIT) and continuous moderate-intensity training (MICT) on people with PD, with HIIT producing better results [104].

AET also improves motor-learning ability by enhancing the plasticity of motor-related structures [98]. Different types of exercise selectively affect different brain regions and functions. AET has been shown to positively impact the superior temporal and parietal prefrontal cortex and transverse tracts between the frontal and parietal lobes, and thus can improve executive function in PD [105].

Trials with MICT reported an improvement in attention and memory, restored processing speeds, and better performances on verbal fluency and spatial working memory, while long-term and high-frequency training alongside specific training such as tango and treadmill training produced better improvements in cognition. PD patients who participated in the treadmill training (TT) + virtual reality (VR) targeting motor and cognitive aspects of the safe ambulation arm had lower activation than the TT arm in Brodmann area 10 and the inferior frontal gyrus, while the TT arm had lower activation than TT + VR in the cerebellum and middle temporal regions. This reduced activation is considered a return towards normal values via the enhancement of cognitive network efficiency [106].

One of the most common nonmotor symptoms of PD are sleep disorders. These have a prevalence rate of 40–80% in PD. Pharmacological treatments have been found to have a limited effect on improving sleep quality. Alternative means of treatment such as repetitive transcranial magnetic stimulation have also been proven ineffective [107]. 

Studies have demonstrated the beneficial effect that regular MICT has on sleep quality. Chronic exercise appears to be uniformly beneficial to both older and younger participants; however, its effect on sleep-onset latency in the elderly is weaker. Regular exercise has a limited effect on total sleep time and efficiency, and a moderate effect on sleep latency [101,108]. 

Acute exercise has also been proven to be effective in the treatment of several sleep disorders. It causes a reduction in rapid-eye sleep but has no significant effect on total sleep time, slow-wave sleep, sleep-onset latency and sleep efficiency. Time of exercise is also an important factor. Exercise carried out three-to-eight hours before bedtime yielded the best results. Another important factor is exercise duration, with longer-lasting exercise producing better results [108]. 

##### Treadmill Training 

Patients with PD have difficulties with turning and gait, as shown by the slow turning and freezing of gait (FOG). These motor issues increase the potential risk of falls for PD patients [65]. Exercise has been shown to also reduce motor dysfunction in advanced PD by improving posture, balance control and mobility amongst others. Treadmill training improves gait parameters such as velocity and step length of PD patients. As such it is especially useful for gait training. Studies show that gait performance in relation to walking speed, step length and support time improved after 4 weeks of training, with effects lasting a minimum of 3 months [65]. Treadmill training also increases neuroplasticity, protects dopaminergic neurons and regulates several signaling pathways [109,110].

Turning-based training on a treadmill benefits the vestibular integration ability of PD patients. Vestibular input is essential to prepare the body for turning, and vestibular feedback assists control of axial movements when whole-body rotation takes place [111]. 

Reduced muscle strength and deficiencies in functional mobility such as balance ability also influences gait performance and turning performance. As such, a specific exercise program designed to improve balance, strengthen muscles and improve vestibular functions is necessary for patients with PD [111]. 

##### Body Weight–Supported Treadmill Training

BWSTT is an option for PD patients who for a number of reasons, such as severe postural instability or orthostatic hypotension, are unable to participate in traditional ground gait training.

Body weight–supported treadmill training is generally well-tolerated, but special caution should be taken with patients who suffer from anxiety and chronic pain [112]. 

BWSTT has been documented to be a promising rehabilitative method for improving gait impairment, balance dysfunction and postural instability in patients with PD [99,113].

##### Robot-Assisted Gait Training

Robot training is another safe and realistic mode of rehabilitation therapy for mild PD patients. Robot-assisted gait therapy (RAGT) could enhance bradykinesia, leg agility, gait, posture and motivation, while reducing freezing, rigidity, gait, leg agility and posture in PD patients, per an assessment using the Unified Parkinson’s Disease Rating Scale [114]. It has been demonstrated that RAGT is more effective in regards to improving gait kinematics in comparison with intensive treadmill therapy [115]. 

In summary, RAGT is an effective therapy program for improving gait dysfunctions in PD patients. 

##### Virtual Reality

Virtual reality (VR) used as an addition to physical exercise provides visual, somatosensory and auditory stimulation needed to improve the gait function of PD patients [99,116].

The possibility of real-time multiple sensory interactions thanks to the simulation of different virtual environments of real and daily life tasks helps to promote task changes and enhance motivation and motor-learning ability during rehabilitation therapy [117]. 

VR enables dual-tasking training and requires patients to simultaneously coordinate the conducting of attention transfer, information processing, sensory integration and motion planning [118].

##### Balance Training

Balance-training exercises are exercises that challenge a person to control the center of gravity of the body during destabilizing movements and/or reduce the size of a person’s support base. It has been documented that balance and gait training positively impact balance and gait function in PD patients and reduce their short- and long-term fall rates [119]. It is thought that improved sensory integration resulting in enhanced posture control in PD patients can be achieved via balance training [120]. Balance is a progressive and highly challenging balance-training program aimed at treating balance-control dysfunction in mild-to-moderate PD patients [121]. It has been shown to markedly boost gait and balance function [122]. The training benefits of Hibalance decreased within six months following the intervention, indicating that regular repetition of the therapy is most likely required to maintain its therapeutic effect [123]. 

Another means of intensive balance training and motor rehabilitation in PD patients is via the use of computer games (exercise + games = exergames) [124]. Exergames designed specifically for PD deficits may help increase player motivation, enjoyment and efficiency [125]. Exergames provide a better therapeutic effect in comparison with monotherapy due to the combination of cognitive therapy and motor training during the use of this form of training [126]. 

##### Progressive-Resistance Training

Progressive-resistance training (PRT) is a strength training method that involves the use of a small number of repetitive motions in small numbers till fatigue while allowing for sufficient recovery between repetitions and steadily increasing the resistance as the ability of muscles to generate force increases [127]. 

It is also used as a complementary therapy to improve sleep quality in PD patients [128]. 

The incorporation of other types of exercise training such as balance training to PRT furthermore reduces postural instability, and in effect improves quality of life in patients with PD [129]. 

High-speed resistance training effectively reduces the bradykinesia score in limbs measured by UPDRS motor assessment, and increases muscular strength in patients with mild-to-moderate PD [63]. 

##### Complementary Exercise

Non-conventional strategies that have been used in the treatment of PD patients include music and dance therapy, as well as martial arts such as tai chi.

##### Tango

While learning the Tango, each participant has to pay adequate attention to the other participant’s movements and general body coordination [130]. As such, Tango could improve spatial cognition in patients with PD due to the necessity of recalling spatial postures while learning the dance. This information then needs to be stored for later use and as such additionally improves cognitive ability [131]. 

The dance can also enhance one’s sense of wellbeing [132]. 

In summary, Tango can aid the treatment of emotional disorders, cognitive impairment and mobility disorders in PD patients.

##### Qigong

Qigong is a Chinese exercise technique that involves the use of meditation, coordinated movement exercises and controlled breathing [133]. Deficiencies in knee extension and heel stride could be corrected by the precise movements of the lower extremities during the practice of Qigong [134,135]. This form of exercise could improve muscle rigidity and timed up and go (TUG) test scores, as well as balance and hand–eye coordination. These potential beneficial effects make Qigong a useful form of rehabilitation for patients with PD [134]. A study by Liu et al. demonstrated that the practice of Baduanjin, which is a type of Qigong, provoked a positive improvement in functional mobility, gait outcome and sleep quality in elderly PD patients [134]. 

In conclusion, it can be said that an alleviation of balance dysfunction, sleep and gait disturbances can be achieved via implementation of Qigong exercise. 

##### Tai Chi

Tai chi is a Chinese martial art composed of a series of gentle, slow physical movements linked together in a sequence ensuring a continuous flow of motion. Benefits resulting from the practice of tai chi include improved flexibility, posture, mental concentration and general wellbeing [136,137].

One study observed an improvement in dynamic postural stability in patients in the mild and moderate stages of PD [138]. Another study, which lasted 16 weeks, showed enhanced psychological wellbeing and improved cognitive function in PD patients [139]. 

The use of tai chi training was shown to reduce dyskinesia by improving the ability of PD patients to implement effective sway strategies and carry out precise movements with improved balance control. This means that patients are more likely to effectively perform normal day-to-day activities such as reaching for objects placed higher, switching from a sitting position to standing (or vice versa) and walking with a lower risk of falls. Improved gait velocity in these participants also led to increases in stride length. As such, tai chi would appear to be an effective exercise intervention for the improvement of postural stability and functional ability in people with Parkinson’s disease [137].

##### Yoga

Yoga is an ancient philosophy that originated from India and is a collection of mental and physical practices aimed at creating harmony between mind and body. The physical practice involves a series of exercise movements that activate the stretch receptors in muscles, ligaments and joints, and as such improve flexibility and strength [140,141]. As a result of mindfulness exercises, yoga practitioners are able to reevaluate feelings of pain, difficulty or disability, thereby improving one’s acceptance of disability and further functioning despite the physical limitations associated with disability [142]. Several studies have demonstrated that regular practice of yoga is able to improve balance, agility, physical alignment, strength, flexibility, mental and emotional health, as well as general wellbeing [140,141,143] The comparatively low cost of yoga therapy, as well as its minimal side effects, make it a highly viable option for patients experiencing continual loss of equilibrium resulting from neurodegeneration associated with PD while attempting to remain active in daily activities. Yoga programs involving mindfulness could be effective in the alleviation of psychological distress and improvement of physical wellbeing, and therefore, enhanced HRQoL [142]. 

##### Summary

In conclusion, there is a large variety of possible rehabilitative interventions in the course of PD therapy. These approaches include general physiotherapy, e.g., stretching, balance and postural exercises; aerobic activities such as treadmill training; and occupational therapy. All of the aforementioned approaches are often implemented to improve varied aspects relating to mobility [144].

There is a large amount of research supporting the notion that the risk of PD development can be effectively lowered by exercise. In general, a variety of exercise-therapy methods have been noted to exert a therapeutic effect on PD-related disorders, both motor and nonmotor. The effects of aerobic exercise are among those that have been most-investigated to date; these range from an improved quality of life to enhanced motor and cognitive functions. Newer treatment approaches such as VR technology have been found to react to visual, auditory and somatosensory stimulation in patients with PD [116]. 

It is worth noting that the extent of previously mentioned beneficial effects of exercise are dependent on the type of exercise and also on the intensity and duration of said exercise, with moderate-to-vigorous intensity alongside longer duration periods and a higher frequency of exercise yielding better results. For example, a study by Frazzitta et al. [145] demonstrated that an intensive physiotherapy program involving 2 daily sessions, 5 days a week over a course of 4 weeks produced beneficial effects lasting over 12 months and resulting in LevoDopa dosage reduction. However, another study by Abbruzzese et al. in 2014 demonstrated a different outcome in regard to the beneficial effects of exercise intensity. Patients in low or intermediate frequency groups (sessions held twice and thrice weekly) showed significant improvement and longer lasting benefits (lasting at least 24 months) in comparison with patients who had sessions 5 times a week. Such different results further buttress the need for more research in this area [146]. 

Exercise-related treatment programs for patients with PD should actively aim at improving core areas affected by the disease such as balance and gait and manual and physical capabilities. Thus, such programs should be tailored specifically to the needs of each patient and to the stage of disease progression [144]. 

#### 4.2.2. Cognitive PD Rehabilitation

In PD dementia, the cognitive symptom profile is usually milder compared to AD; the cognitive problems begin in the later disease phase and concern more executive-function disorders. The problems of memory and learning new information are often secondary to the issues related to executive functions [147]. The most common psychological methods applied are cognitive training, dual-task training (cognitive and physical combined simultaneously) and CBT for comorbidities such as mood and anxiety disorders [148]. 

On the basis of the current knowledge concerning the psychological methods supporting cognitive functioning in PD patients, it seems difficult to draw any reliable conclusions. For example, one of the studies suggested that the efficacy of cognitive rehabilitation programs is improving cognition [149]. The most popular therapeutic methods are centered around computer-based training of memory, attention, language and executive functions [149]. In other studies, research shows that there is no reliable evidence that cognitive training is helpful for people with PD dementia. It has been mentioned that cognitive training may activate mechanisms of cerebral plasticity and slow PD-associated cognitive decline, but future studies should investigate the potential mechanisms of cognitive training [150]. Some of the most interesting methods supporting PD patients might be dual-task training, combining motor and cognitive training. The results show that improving gait and executive function simultaneously might be very useful for PD dementia patients and may lead to significant improvements in velocity and length stride, as well as in the quality of life [151]. 

In terms of emotional problems in PD, the most effective seem to be anxiety and depression CBT protocols [152,153] performed independently or combined with pharmacotherapy [154]. Most of the interventions already reported took place in groups or were telephone-based [152]. CBT could help in reducing anxiety [155], negative thoughts [152], psychological distress [156] and somatic complaints [154]. Additionally, an improvement can be observed in a diverse array of depressive symptoms [154] as well as treatment satisfaction and acceptability [155]. 

### 4.3. Amyotrophic Lateral Sclerosis 

ALS is a disease that leads to paralysis as a result of progressive deterioration and loss of function of motor neurons in the brain and spinal cord.

The two types of AML with variations based on genetics are familiar and sporadic (also known as idiopathic). Symptoms are described as “limb onset” which includes difficulty with actions such as holding a cup, buttoning shirts, changes in running/walking gaits; and “bulbar onset” involving difficulties with chewing, swallowing and impaired speech. Diagnosis is based on results of clinical examination alongside nerve conduction studies, EMG and laboratory tests. Criteria developed by El Escorial World Federation of Neurology in 1998, revised El Escorial criteria 2015 and Awaji Island criteria are also useful in the diagnosis of ALS. The use of Awaji Island criteria has greatly improved the diagnosis of ALS, particularly in regards to the bulbar-onset form [157]. This is because the Awaji criteria developed EMG measures that facilitate diagnosis based on the occurrence of fasciculations alongside chronic neuropathic muscle potentials or fibrillation muscle potentials as markers of acute denervation in patients with clinically suspected ALS [158]. The ALSFRS was used in the assessment of activities of daily living in ALS patients. It has since been revised and renamed the ALSFRS-R, with the main difference being that ALSFRS-R additionally assesses respiratory function along with bulbar and limb function, thus increasing the sensitivity of the scale [158]. Assessment of quality of life is best carried out using the ALSSQOL-R questionnaire. Use of the aforementioned scales is advised in both clinical practice and research [159].

Studies have been carried out assessing the effect that endurance and resistance training have on symptoms and on the general health of ALS patients. Regardless of its modality, exercise seems to have beneficial effects on the quality of life of ALS patients, but its impact on survival rates is yet to be confirmed. To date, there have been no negative outcomes discovered in regard to disease progression as a result of introducing an exercise regimen [160]. While much is known about the positive effects that endurance training has on respiratory capacity, exercise tolerance and physical performance in ALS patients, there is still a need for further studies encompassing a larger group of participants and with the inclusion of a control group. These studies could look into creating an optimal endurance training protocol for ALS patients. Resistance exercise builds muscle strength, stimulates muscle growth and enables the proper maintenance of skeletal muscle function [161]. These benefits have also been observed in ALS patients. One of the first published reports of resistance training in ALS patients concluded that resistance exercise involving the upper extremities increases muscle static strength of the upper body [162]. A later study further supported observations regarding the positive, albeit temporary, effect that consistent resistance exercise has on slowing down motor function deficits in patients with ALS [160].

While the recent evidence supports the claim that there are several benefits of resistance/endurance exercise, including enhancing the quality of life of ALS sufferers, there have been none noted so far regarding life expectancy. Furthermore, the effect of exercise in the alleviation of sleep disorders in ALS has also not been noted. As such, more research is crucial to assess the possible role of exercise in ALS-related sleep disorder treatment [84]. 

Until recently, the role of physical exercise in ALS pathology has been controversial. Some epidemiological studies suggested that people practicing intense physical activity, such as professional soccer or football players, were at a higher risk of developing the disease [163]. Physical exercise and activity has been positively correlated with ALS incidence in a number of studies. Despite this, a close relationship between physical exercise and ALS has not been shown, and it has been suggested that the increased risk is not due to actual physical activity itself, but instead is due to unknown congenital factors predisposing individuals to physical activity and fitness. It has been proposed that it is this “athletic phenotype” that carries an increased risk of ALS. There are several plausible explanations for how exercise directly could cause ALS, including increased oxidative stress and potentiation of environmental toxins [164]. 

Regular, moderate exercise over a period of 6 months has been shown to slow down the rate of motor function loss, exhaustion and pain in ALS patients. Daily stretching and resistance exercises also improved quality of life in these patients by enhancing motor function [165]. 

However, these studies have been insufficient in undoubtedly proving the hypothesis that moderate physical activity is beneficial for ALS patients. This limitation results from the small number of study participants and the short duration period of the study.

Future studies are essential to ascertain the benefits or harmful effects of exercise on disease progression [163].

As a consequence of ALS, many cognitive (executive, attention and memory) function problems, as well as emotional lability (in the case of pseudobulbar affect), are observed in 35–55% of individuals [166]. In 10–15% of patients, cognitive problems are severe enough to meet frontotemporal dementia (FTD) diagnosis. The symptoms manifest as changes in behavior and executive and language impairment [167]. There are just a few reports focusing on psychotherapeutic methods of treating emotional problems during ALS. Initial reports suggest that the most effective methods are CBT and MBTs as they reduce depressive and anxiety symptoms and improve quality of life [168,169].

### 4.4. Huntington’s Disease

HD is a progressive, neurodegenerative disease that is typically inherited in an autosomal dominant manner. Symptoms include chorea, spasticity, rigidity, bradykinesia and akathisia resulting from progressive motor dysfunction. Other symptoms include significant sleep disorders, impaired cognitive abilities and neuropsychiatric symptoms such as anxiety and depression. 

HD diagnosis is contingent on a positive family history or positive genetic test alongside the occurrence of motor disturbance as defined by the UHDRS TMS diagnostic confidence score [170].

The UHDRS has been further expanded to include the following scales: UHDRS-TFC, UHDRS-FAS and UHDRS-IS. Other scales include ABC, which is a patient-completed scale evaluating balance confidence and fear of falls; RMI, which is recommended for use in the assessment of severity of mobility restriction; TMT, which assesses gait and balance in patients with up to stage 3 HD and for screening for risk of falls; BBS, used in the assessment of balance impairment severity in ambulatory HD, and which can also be helpful in assessing fall risks; and the Six-Minute Walk test, recommended for use in assessing walking endurance [171].

While there is currently no cure available, exercise training, with its potential ability to improve cognitive and motor function in HD patients, should be considered a viable and safe treatment option [81].

The optimal exercise intervention would be one acceptable to a large amount of HD patients, taking into consideration personal preferences including those relating to exercise location, as this would increase willingness to participate in such activities as well as adherence. 

These exercise programs also need to be closely monitored, and if needed, modified due to the high incidence of cardiac events in patients with HD [172].

Studies assessing different exercise programs and their effects have found that both aerobic and resistive training exercises are safe and provide benefits when performed 3–4 times a week for a period of at least 12 weeks, with each session lasting 30–50 min at moderate intensity [173]. 

More studies researching the long-term effects in HD patients are essential.

Two controlled and randomized trials examined the role of exercise in the alleviation of respiratory dysfunction. Modest improvements were noticed in regards to pulmonary function measures, dyspnea, cough effectiveness, swallowing and walking endurance after the introduction of resistive respiratory-muscle training [174,175]. The improvements observed, such as increased maximum inspiratory and expiratory pressures and increased forced vital capacity occurred at a much higher rate in the training group than the control group [174]. These results suggest that resistive respiratory-muscle training could be of benefit to HD persons, but more studies involving larger groups of participants are needed to determine the best training. Larger studies are needed to determine the best training patterns for optimal pulmonary function [173]. Available studies unanimously state that exercise training for HD patients is feasible. While most of these studies concentrated on patients in the early-to-mid stages of the disease, there are also studies indicating the feasibility of exercise in the late stage HD [176,177].

There is evidence to support the use of assistive devices such as walkers and canes, modified seating arrangements and caregiver training to enable safety while walking for patients in the later stages of HD [178]. 

Four-wheeled walkers with frontal swivel castors were shown to provide superior stability and safety during walking in straight lines and while turning, in comparison with other assistive devices. These walkers induced the best physiological gait pattern [179,180].

There is inconclusive evidence to support the use of aqua therapy in HD currently [181]. Evidence supporting the use of rehabilitative interventions targeting corticobulbar symptoms in HD patients is also inconclusive. Available studies suggesting possible short-term motor function improvements following intensive rehabilitation therapy were limited by the likelihood of risk of bias and insufficient use of reliable, objective tools. However, the existence of preliminary evidence justifies the necessity of more research in this field [182]. 

Up to 90% of HD patients experience a variety of sleep disorders. These sleep disturbances include insomnia, daytime sleepiness and frequent awakenings. Sleep disorders in HD have been found to exacerbate depression, anxiety and disinhibition in affected patients when compared to patients without sleep disturbances. 

There are few treatments currently available for sleep disorders in HD. While there are several studies regarding the possibility of exercise interventions as a treatment for motor dysfunction in HD, there is limited information in regards to the effect of exercise on HD-related sleep disorders. More studies are required to properly assess the impact of exercise on sleep and the circadian cycle in HD [84]. 

Studies show that resistance training could potentially increase body mass in patients in early- and mid-stage HD. Additionally, proper nutritional advice is a necessity to avoid the loss of skeletal muscle mass as a result of insufficient energy and protein consumption. 

Physical activity and cardiovascular fitness are known to reduce cardiovascular mortality in the general population; therefore, it is reasonable to assume that a similar effect can be expected in HD patients. 

The positive effect of exercise on motor function has been proven in a majority of studies carried out; however, it is difficult to assess the effect of exercise on the UHDRS motor score due to variations in the progression of motor scores in control groups. 

Current results of research on the effects of exercise training on motor function show that exercise interventions can delay disease progression in HD patients. It is undetermined if this delay improves the capability of patients to carry out daily life activities. Prolonged interventions are necessary to evaluate this issue. 

It is becoming increasingly apparent that rehabilitation therapy could become a highly useful tool in managing HD. This information is of particular relevance to patients with early PD when certain neurological changes may still be reversible [183]. 

Treating motor impairments resulting from neurodegeneration could potentially delay disease progression and maximize functional abilities in the long term. The benefits of these interventions could be further boosted by expediting adaptive neuroplasticity when used alongside disease-modifying drugs or cell-replacement therapy [180].

In HD, a lot of neuropsychiatric symptoms such as executive, attention, memory impairment and emotional or behavioral control problems (for example, aggression) or mood disorders are often observed [184]. There is still very little evidence concerning the psychological methods of supporting HD patients. It is now known that memory-oriented cognitive rehabilitation intervention should be provided for patients with HD to improve daily functioning [185]. Few reports suggest using internal memory strategies (e.g., making associations and visual imagery), and applying external memory aids to remind the individual of the specific details of real-life tasks in cognitive programs [185]. Memory and control-oriented cognitive rehabilitation intervention could be provided for patients with HD to improve daily functioning [185]. For example, a multidisciplinary 1-year cognitive rehabilitation program developed by [186] had an impact on memory, attention, and executive function maintenance, despite the deterioration of the general cognitive indicator during that time.

In emotional control aspects, there are preliminary reports about a significant reduction in the levels of aggression after behavioral therapy [187]. In turn, there are no significant results concerning the impact of CBT on psychological stress or mood disorders reduction as yet [156].

### 4.5. Wilson Disease

WD presents itself in a variety of ways, which include hepatic, neurological, ophthalmological and psychiatric manifestations. Kayser–Fleischer rings and sunflower cataracts are ophthalmological symptoms. WD often presents itself with psychiatric manifestations. 20–60% of WD patients eventually develop depression. Other psychiatric manifestations include emotional lability, social disinhibition, irritability, aggression, hypersexuality, lack of criticism and inability to predict social consequences. These could be as a result of frontal-lobe or frontal-lobe-pathway lesions. One common neurological symptom is tremors, which can present as resting, postural or kinetic tremors. Tremors can be unilateral or bilateral and most often involve distal parts of the upper extremities. Involuntary movements similar to tremors affecting the hands, known as asterixis or flapping tremors, are also observed in patients with hepatic encephalopathy and liver failure. Other neurological symptoms of WD are dystonia, bradykinesia, rigidity, hyposmia, gait and postural disorders, as well as dysarthria, dysphagia and drooling. Clinical scales such as UWDRS and Global Assessment Scale for WD used to assess neurological deficits and functional impairments were developed as a result of the wide heterogeneity of neurological symptoms associated with WD. Currently, brain MRI is the most important neuroradiological diagnostic tool; it is also potentially helpful in the monitoring of treatment.

Due to the wide range of symptoms that patients with WD present with, a combination of clinical features and laboratory parameters are essential to establish diagnosis in most cases.

In order to overcome this diagnostic difficulty, the Leipzig score is used. It is calculated using certain clinical signs such as Kayser–Fleischer rings, and neurologic symptoms as well as laboratory findings, e.g., copper levels in serum, urine and liver serum ceruloplasmin levels and genetic testing, are scored from 0 (meaning absent) to 2 (present). A score of 4 or more indicates a high probability of a WD diagnosis [188].

Ti date there is still limited research addressing the role of physiotherapy in the treatment of Wilson’s disease and PKAN. In 2016, Maiarú M. et al. wrote about methods of physiotherapy used in the treatment of a single patient with Wilson’s disease. Treatment aims included fall-risk reduction and reduced difficulty in overcoming architectural barriers. Balance exercises—both static and dynamic, as well as functional capacity training—were the foundation of the treatment. Exercises were carried out twice a week over a period of 2 months and 20 days alongside home exercises performed thrice a week [189]. 

#### 4.5.1. Exercises Aimed at Improving Static Balance

These included standing exercises carried out on a stable surface with feet held together and gradually working up to balancing on one foot. During these exercises, patients had their eyes closed in order to improve reception of somatosensory information. They were performed in a series of four 1-minute sets with 3-minute breaks in between sets. 

#### 4.5.2. Exercises Aimed at Improving Dynamic Balance

These exercises took place on a treadmill under the supervision of a kinesiologist. As the patient was initially unable to cope with the pace without the use of his upper limbs, the first goal of training was attaining the required stability. Once the patient was sufficiently stable, horizontal and vertical head turns were introduced. Later, the patient was able to carry out the exercises on the treadmill with his eyes closed. Exercises were performed in sets lasting 3 min with 5 min breaks in between sets over the period of an hour per session. 

#### 4.5.3. Exercises Targeting Functional Capacity Training

The stage aimed to alleviate the difficulties that patient had in carrying out everyday activities, such as walking up and down stairs or picking objects off the floor. Exercise sets lasted as long as the patient was able to tolerate with 3–5 min breaks between sets, over a 30 min period. These sessions were followed up with 15 min lower limb-stretching sessions. No injuries were noted over the course of this training and the patient also reported his satisfaction with the program. 

During the final evaluation, a self-reported improvement in balance confidence was reported by the patient. This improvement was also objectively demonstrated by results of TUG, 10MWT, FGA and CTSIB-M tests. 

Post-treatment, the difference noted between the normal gait speed and maximum possible gait speed shows that the patient gained the ability to vary his speed extensively. This could be used as a marker assessing the potential for adaption to varied environments and task demands [189]. 

Physiotherapy and occupational therapy are useful in the treatment of neurological symptoms associated with this disease.

As it can take up to half a year for copper-chelating treatment to start working, these forms of therapy could aid in the reduction in symptoms such as ataxia, dystonia and tremors while also diminishing the risk of contractures resulting from dystonia. 

Genetic counseling carried out by a genetic nurse and clinician geneticist is essential in the education of patients in order to prevent transmission of the mutated gene. 

Mortality and morbidity of the disease can only be reduced via a combination of the approaches mentioned above [190].

#### 4.5.4. Therapy Targeting Dysphagia

50% of patients with Wilson’s disease suffer from dysphagia, which has a debilitating impact on patients’ quality of life in the later stages of the disease. Da Silva-Junior et al. reported that dysphagia in patients with Wilson’s disease was caused by pre- and postsynaptic dopamine deficiency resulting from the accumulation of copper in the brain. As such, similarities were observed between dysphagia in Wilson’s disease and dysphagia in Parkinson’s disease [191]. 

This led to scintigraphic tests further assessing these similarities being carried out. Dysphagia caused by Wilson’s disease was found to be characterized by extended oral transit time, an increased percentage of oral and pharyngeal residue, as well as prolonged pharyngeal transit time in comparison to that observed in healthy individuals of a similar age [191]. Other factors that contribute to difficulty in bolus formation and prolonged oral transit time include rigidity, bradykinesia and tremors involving the tongue and general oral musculature. There is also an increased risk of aspiration due to misdirected swallows resulting from pharyngeal motility disorders. It has been shown that this type of dysphagia can be alleviated with the use of NMES [192]. 

## 5. Conclusions

It has been proven that the use of prevention measures such as physical activity is essential in order to maintain proper motor function and stave off impairments that result from mobility restrictions in patients suffering from different neurological conditions [178].

Taking into consideration the immense potential effect that exercise could have in regards to disease modification and general health improvement, there is an urgent need for more research to be conducted to further examine these benefits [180].

Most recent data available indicate that cognitive stimulation and cognitive training could foster independence and maintenance of quality of life in patients with AD [88]. In the case of PD, the actual evidence remains inconclusive, but a new form of training—especially dual-task training—could be helpful in improving gait and quality of life [151]. Additionally, CBT could be effective in the treatment of depression and anxiety disorders in PD patients [154]. In terms of HD, some of the reports have shown that memory training could be helpful in maintaining cognitive functioning [185] and behavioral training could lead to reduced aggression [187]. Finally, in patients with ALS, after CBT and MBT, depression- and anxiety-symptom reduction was achieved and quality of life gradually improved [168,169].

The complex range of symptoms related to neurodegenerative disorders that benefit from multidisciplinary care emphasize the importance of conducting research that serves to highlight the most effective rehabilitation modalities for this patient population. The goals of the study should include establishment of safety and tolerability of exercise programs in order to assess their effect on function. Other goals include defining the impact of commonly deployed rehabilitation interventions, such as bracing, patient safety and independence. 

There is also an urgent need to document the impact of rehabilitation on the ability to perform desired activities despite expected disease progression via a collection of patient-centered outcome data. The traditional model for rehabilitation is based on the principle of improvement; however, improvement is normally measured in terms of the perceived amount of change in impairments such as strength, physical capacity and improved walking efficiency. This model, however, cannot be applied to progressive diseases such as neurodegenerative disorders, as an improved neurological state is not expected. It is also a common experience that rehabilitation interventions can have an immense effect on the performance levels of certain activities despite persistent neurological symptoms. For example, the use of assistive devices and training on proper transfer techniques with appropriate modifications for disease progression may allow patients to get out of bed safely and efficiently, leave their homes, work and be active in their communities. Thus, collecting prospective patient-centered outcome data may help document the positive impact of rehabilitation on activity limitations and participation restrictions, hence providing evidence regarding the importance of rehabilitation in neurodegenerative disorders. Future studies should concentrate on large-scale trials of clinical effectiveness versus control–comparison intervention or effectiveness of cognitive training against other active or social interventions. It will be important to examine whether any effects observed are generalized to everyday functions and tasks of daily living. The literature on the subject does not provide sufficient insights regarding the emotional treatment protocol in many neuropsychiatric disorders. Additionally, future studies should focus on testing whether the type, length, kind of exercises and frequency of intervention impact the obtained efficacy results. Of particular importance seems to be the need for conducting further research into the long-term effects of the discussed interventions.

### Limitations and Future Directions

This article has several limitations. The aforementioned diseases occur with varying degrees of frequency and some are extremely rare. During our research we were unable to find any information concerning the role of rehabilitation in NBIA and aceruloplaminemia. Only one study describing the rehabilitation of a patient with WD was noted. The variations in length of studies, disease types and diagnostic criteria mean a specific rehabilitation program for any of the aforementioned diseases is yet to be established. Exercise training seems to be safe and feasible for patients with neurodegenerative disorders; however, current knowledge is based on short, small-scale studies and cannot be applied to all neurodegenerative diseases. Therefore, longer-term interventions with larger numbers of cohorts are necessary to draw firm conclusions about the potentially beneficial effects of exercise training in neurodegenerative disorders. 

## Data Availability

Not applicable.

## Supplementary Materials

The following supporting information can be downloaded at: https://www.mdpi.com/article/10.3390/brainsci12030345/s1, Table S1: Examples of exercises in neurodegenerative diseases.
