# Supplementary material for: Cognitive and Physical Intervention in Metals’ Dysfunction and Neurodegeneration
Source: Brain Sci. 2022 Mar 3;12(3):345. doi: 10.3390/brainsci12030345 (PMC8946530; doi:10.3390/brainsci12030345)
Supplement: Supplementary file 1 [file brainsci-12-00345-s001.zip › brainsci-1590257-supplementary.pdf]

Tabel S1. Examples of exercises in neurodegenerative diseases.

Disease	Examples of exercises	
	Physical	Neuropsychological
ALZHEIMER'S DISEASE	there are no clear health instructions about physical activity	reminiscence therapy, special cognitive rehabilitation program and cognitive training
PARKINSON DISEASE	aerobic exercise training (AET), treadmill training, body weight–supported treadmill training (BWSTT), robot training - robot-assisted gait therapy (RAGT), virtual reality (VR), balance training, progressive resistance training (PRT), complementary exercise (tango, qigong, tai chi, yoga)	cognitive training, dual-task training (cognitive and physical, combining executive at the same time) and CBT for comorbidities such as mood and anxiety disorders
ALS	regular, moderate, daily stretching and resistance exercises	CBT and MBTs
HUNTINGTON'S DISEASE	aerobic and resistive training exercises	behavioral therapy, CBT
WILSON DISEASE	exercises aimed at improving static and dynamic balance	there are no clear health instructions about neuropsychological therapy
